# Plurilingualism as a Catalyst for Creativity in Superdiverse Societies: A Systemic Analysis

**DOI:** 10.3389/fpsyg.2017.02169

**Published:** 2017-12-22

**Authors:** Enrica Piccardo

**Affiliations:** ^1^Ontario Institute for Studies in Education, University of Toronto, Toronto, ON, Canada; ^2^ESPE, Université Grenoble Alpes, Grenoble, France

**Keywords:** plurilingualism, landscape of affordances, Dynamic System Theory, creativity, plurilanguaging

## Abstract

Post-industrial societies are characterized by a high degree of mobility which manifests itself through waves of migration and affects all knowledge domains and all aspects of both individual and collective lives. This situation presents challenges under the pressure of a powerfully uniformizing globalization. However, the exponential increase of diversity linked to intensified mobility is also conducive to social transformations since, when the numerous languages and cultures of the migrants encounter the languages and cultures of the host countries, they act as catalyzers of change. This article considers such social transformation in the light of the concept of plurilingualism as distinct from multilingualism, explaining the advantages of the former over the latter in such contexts, and analyzes possible synergies between plurilingualism and creativity through the lens of complexity theories and the theory of affordances, with the related concepts of ‘affordance spaces’ and landscape of affordances. After a brief introduction of the main tenets of complexity theories and affordances, the article builds on three complementary models of creativity, using complexity theories as a framework and discusses the specific characteristics and potential of plurilingualism by explaining how it can transform diversity from an obstacle into an opportunity, a possibility for action. The triadic relationship between creativity, plurilingualism, and complexity is considered. As a result, the article suggests that plurilingualism can create conditions conducive to creativity thanks to its multiple and flexible nature that values all forms of cross-fertilization and the uniqueness of the resulting individual trajectories. Without claiming any causal relationship between plurilingualism and creativity, the paper explains the reasons why it is crucial to nurture and foster plurilingualism in order to provide favorable conditions for creativity and change. The article explains the characteristics and implications of plurilanguaging, and the potential for individuals to embrace a holistic, complex view of languages and cultures and to experience empowerment in the process of perceiving and exploring linguistic and cultural diversity, hybridity and interconnections, thus discovering and liberating their full creative repertoire.

## Introduction

We live in “a highly dynamic social tapestry” (OECD, 2000, p. 8) despite the ubiquitousness of identical brands. Our societies are increasingly destructured and characterized by phenomena of deterritorialization and reterritorialization (Defert, 2012). The increasing difficulty in tracing neatly delineated homogeneous spaces is one of the main signs of liquid modernity (Bauman, 2000). In liquid societies frontiers become increasingly volatile and an exponential increase in diversity translates into change to cultural and linguistic forms. The reaction vis-à-vis these changes situates itself along a continuum going from acceptance, often in the form of active interest in diversity, as opposed to rejection, with folkloric retention of simulacres of difference, recalling findings in research on exposure to multicultural experiences (Chao et al., 2015).

Accepting multiplication of diversity is challenging. It confronts a deeply rooted western quest for universal examples and neat categorization as part of a penchant for analytic, linear Cartesian vision. Thus, the move from a ‘solid’ to a ‘liquid’ conceptualization of the world implies a daunting shift from a paradigm of simplicity to one of complexity (Morin, 1990/2005), which requires time, commitment and the ability to deal with uncertainty and phases of chaos.

In education, diversity takes the form of students arriving with a life trajectory characterized by a plurality of languages and culture, which is seen either as a problem or as an asset. Language is in the front line as the languages and cultures of migrants interact with the languages and cultures of host societies, becoming potential catalysts of change (Piccardo, 2013). Simultaneously, western countries are moving from industrial economies to knowledge-based economies (European Commission, 2009), where politicians, educators, and business leaders all increasingly realize that creativity and innovation are prerequisites for economic success (Sawyer, 2012).

In this context, interest in the relationship between proficiency in more than one language and cognition has increased considerably, with a particular focus on a possible cognitive advantage of bilinguals over monolinguals. In addition, increasingly articulated models are being proposed to investigate the creative process. These developments suggest challenging questions: What characteristics and vision of linguistic diversity align with creativity and could potentially foster it? What theoretical framework(s) might help us cast light onto such an elusive connection? Finding answers to these questions requires an interdisciplinary perspective including advances in different scientific domains, from psychology and neurosciences to linguistics and education. This article provides some reflections on these questions by referring to complexity theories and employing both Dynamic System Theory (DST) and the concept of affordances as lenses through which to investigate both creativity and plurilingualism. After presenting a theoretical framework which combines DST and affordances, the difference between multilingualism and plurilingualism is clarified, and relevant models of creativity are discussed from a plurilingual perspective. The article then analyzes the way in which both plurilingualism and the new vision of creativity that these models support align with complexity theories and the notion of affordances. Finally, potential synergies between plurilingualism and creativity are discussed from a systemic perspective. As this article investigates relationships and synergies between four main concepts, plurilingualism, creativity, complexity, and affordances, these concepts are briefly defined first to help the reader navigate the development of the argument. Each of them is then discussed separately in relation to diversity in order to highlight their interrelation.

## From a Linear to a Complex Vision: Introducing Plurilingualism, Creativity and Affordances

### Plurilingualism

In applied linguistics and language education, reflection is ongoing on the best way to deal with increasing linguistic and cultural diversity. However, the widely used term, multilingualism, does not capture the complexity of such diversity. For this reason, the term plurilingualism (Council of Europe, 1996, 2001; Coste et al., 1997/2009), has been conceptualized in a way that aligns with a complex vision of language education and use. Plurilingualism differs from multilingualism (the simple addition of languages in societies and/or individuals) in that it focuses on the relationships between the languages an individual speaks, the underlying linguistic mechanisms and cultural connotations, the personal linguistic and cultural trajectory as well as the persons’ attitude toward language diversity, stressing openness, curiosity, and flexibility.

### Creativity

Since the complexification of societies inevitably implies increasing ecological and cultural challenges, creativity is a key concept in several domains, from the socio-economic to the scientific. The definition used here encompasses the classic twofold construct of creativity as a capacity to realize a product that is both **novel** and **appropriate/useful** to the context where it appears, as judged by a suitably knowledgeable social group (Barron, 1988; Sternberg and Lubart, 1995; Amabile, 1996; Lubart et al., 2003; Sawyer, 2012) and is complemented by the notion of autonomous, personal reconstruction of concepts and data, free association and disassociation, within a framework of play and pleasure (Piccardo, 2005). The article also considers creativity as a social and individual phenomenon (Sawyer, 2012), in turn building on the distinction between personal and historical creativity proposed earlier by Boden (1992). Not only does this articulated vision of creativity capture both everyday creativity (creativity with a small c) and eminent creativity (creativity with a big C), but it extends this difference to the developmental dimension – giving the ‘Four C’ model of creativity (Kaufman and Beghetto, 2009) – and tackles the potential cross-cultural variations of the construct, adding aesthetics and authenticity to the classic criteria of novelty and utility (Kharkhurin, 2014).

### Affordances

We start from the classical view of affordances as opportunities for action that are offered to individuals (Gibson, 1979/1986) and move toward a broader and more dynamic definition that stresses the interdependence between the environment and individuals (Chemero, 2003) and the notion of co-development in a given cultural context (Glăvenau, 2012).

### Complexity

Dynamic System Theory provides the general conceptual framework of the article. As all three above-mentioned notions refer to phenomena that encompass different elements and can be studied from a variety of perspectives, DST appears to be the fittest for the purpose of situating them in relation to diversity and to highlight their interrelations as it specifically opposes a linear, mechanistic view of phenomena, aiming to capture variation of time and interdependence of factors involved.

The notion of complex system is used here as the main theoretical framework to analyze the conceptual overlap of creativity and plurilingualism. Ecological psychology, particularly the notion of affordances, is then drawn upon in order to explain the role of learners’ agency, i.e., learners’ possibility to act, to take the initiative and thus construct knowledge. This combined theoretical framework helps to highlight the commonalities between plurilingualism and creativity. Since an in-depth discussion of complexity theories is beyond the scope of this article, the focus is given to the notion of complex adaptive systems (CASs), which is at their core. We later move to the notion of affordances, before going back to plurilingualism and creativity.

## Complexity Theories, Dynamic System Theory (DST) and Complex Adaptive Systems (CASs)

Both the terms of complexity theories and DST have been used interchangeably in applied linguistics (Larsen-Freeman and Cameron, 2008) so I will use both terms in this article to refer to the constellation of complexity as a whole, which includes studies on complexity, general system theory, fractals – nested patterns reoccurring in an iterative manner at different scales (Mandelbrot, 1983), and what is known as ‘the butterfly effect.’ I will not describe the differences among these perspectives, but rather focus on the core message that complexity theories convey, and the way they help us reason in terms of CASs when we investigate phenomena difficult to study from a classical, linear perspective.

According to DST, nothing in the universe can be reduced to a mechanical assembly of identifiable components. Everything is complex and therefore impossible to analyze through reductionist approaches. Since the beginning (Weaver, 1948), complexity theories pursued a double movement: (a) a move away from classical physics, with its quantitative data, objectivity and principle of causality, and (b) a focus on ‘organized complexity’ in which elements are in a **non-causal** – but organized – relationship which gives rise to a shape, design, model or pattern. Complexity theories study complex systems, i.e., systems characterized by a high number of interrelated and mutually interactive elements, stress the limitations of investigating problems “as a play of elementary units, the characteristics of which remain unaltered whether they are investigated in isolation or in a complex” (von Bertalanffy, 1950, p. 134) and advocate a united, holistic approach.

von Bertalanffy (1968, p. 39) defined systems as “sets of elements standing in interaction” and underlined openness as their core characteristic, as “every living organism … maintains itself in a continuous inflow and outflow.” Later van Geert (1994, p. 50) defined dynamic systems as “set[s] of variables that mutually affect each other’s changes over time.” Systems started to be seen as self-generating (Varela et al., 1974; Maturana and Varela, 1980). Chaos theory underlined the unpredictability and evolution of systems (Gleick, 1987) and fractal theory (Mandelbrot, 1983) enabled the recognition of a geometric order in phenomena that appear to have no order.

Dynamic System Theory studies variables over time (de Bot, 2016) and systems are increasingly investigated as CAS, where elements generate patterns and collective properties through their interactions. DST has already proved effective in helping the conceptualization of language and language education (De Bot et al., 2007; Larsen-Freeman and Cameron, 2008; van Geert, 2008; Piccardo, 2010, 2015, 2016; Verspoor et al., 2011). Complexity theories also allow modelization of reality (Le Moigne, 1977/1994). Describing, modeling and predicting plays a major role in all scientific work and DST provides powerful metaphors that help satisfy this need (Herdina and Jessner, 2002). DST is particularly suitable for the study of creativity and plurilingualism, as well as of their inter-relationship, since both creativity and plurilingualism include multiple elements, which can be approached from different perspectives and imply a development over time.

Complex adaptive systems are characterized by dynamism, openness, sensitive dependence on initial conditions and non-linearity, self-organization, adaptability and development, and self-similarity. In addition, “[d]ynamic systems are typically nested: large systems consist of subsystems and these in turn consist of sub-subsystems and so on” (de Bot, 2016, p. 126). After a certain time, and independently from initial conditions, CAS evolve toward particular conditions, spaces, or ensembles called **attractors**: “[o]ver time a dynamic system will move from one attractor state to the next, but what the next attractor state will be is unpredictable… and the same system with identical initial conditions may show different attractor states” (de Bot, 2016, p. 128). Attractors go from simple recurring equilibrium states, to growing and changing patterns which represent regions of connected order at the edge of chaos. Finally, CAS are characterized by **phenomena of emergence**, i.e., by the spontaneous development of new properties. We talk about emergent properties to refer to a property of the whole system rather than of any of its isolated components, which emerges from the interaction between these same components. In a similar way, we talk about an emergent process to describe a process that affects the whole without impacting on any of its elementary processes, and that emerges from the interactions and combinations of these elementary processes.

Larsen-Freeman and Cameron (2008) mention several examples of emergence in CAS. A traffic jam is an example of emergence as it is something different from each car that has generated it and can remain for hours while single cars may have passed through it. Another example is a termite nest, where the activity of the termites produces the nest and in turn the shape of the nest impacts the level of activity of the termites. Larsen-Freeman and Cameron (2008, p. 60) say that “[e]mergence in learning occurs when new ideas fall into place” and that the new knowledge acquired influences other ideas. They also point out that a language itself “emerges from the multiple interactions of its speakers” and “learning a language is not a single process of emergence but a succession of cycles of emergence” (Larsen-Freeman and Cameron, 2008, p. 61).

Let us now consider another theory to investigate the point in time immediately before the phenomenon of emergence and the role and action of the two nested CAS (the human being and the environment) in that phase. This is the theory of affordances.

## Affordances

Affordance is a concept first introduced by Gibson (1979/1986), who stresses three principles of the ecological approach:

(a) Perception is direct and not inferential, as was generally thought following Descartes;
(b) Perception is related to action in an interdependent manner; and finally
(c) There is enough information in the environment for both perception and action.

Although Gibson highlighted the dynamic nature of both perception and action, he still viewed affordances as independent from the observer and invariant. Subsequent ecological psychologists as well as cultural and sociocultural psychologists (Cole, 1996; Valsiner and Rosa, 2007) refined and reformulated Gibson’s view of affordances in a way that is particularly relevant for creativity and plurilingualism. Käufer and Chemero (2015, p. 166) clarify that affordances are “opportunities for action in the environment” determined by both the abilities of a subject and the environment. Similarly, Rietveld and Kiverstein (2014, p. 335) stress how “[a]ffordances are relations between aspects of a material environment and abilities available in a form of life.” Using the term ‘situated normativity’ (Rietveld, 2008), they discuss how the concrete situations concerned determine the appropriateness of an individual’s activity, which depends on sociocultural norms. Not only has our environment been sculpted by our sociocultural practices, but also education is a process of learning “to selectively pick up some aspects of the environment while ignoring others” (Rietveld and Kiverstein, 2014, p. 335). Affordances only exist insofar as they can be detected.

There are endless affordances at any given moment but only some invite subjects to act. For Käufer and Chemero (2015), ecological psychology together with DST (what they call “radical embodied cognitive science”) can explain the distinction between affordances and invitations.

“When a human or other animal is engaged in a task, it organizes itself into a temporary, special-purpose dynamical system … and as such it is only sensitive to the affordances that are relevant to that temporary, special-purpose dynamical system. Only those relevant affordances … are experienced as invitations.” (Käufer and Chemero, 2015, p. 203)

**FIGURE 1:**
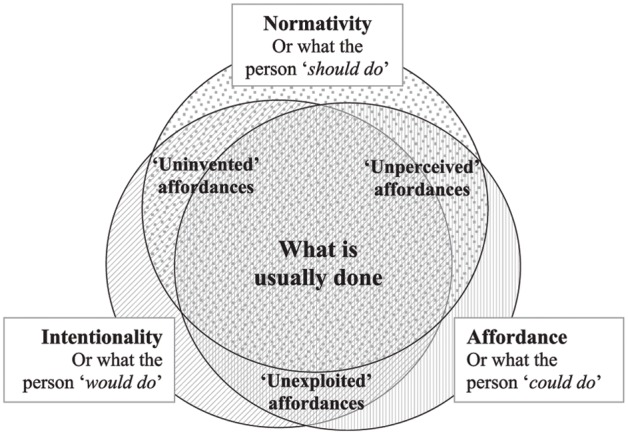
A sociocultural model for an affordance theory of creativity (Glăvenau, 2012, p. 197).

The combination of DST with affordances can explain the concept of ***agency***. As we saw above, agency requires the potential for choice, selection, initiative or decision. However, the way we engage with the environment is determined by culture (Glăvenau, 2012). The world is structured by cognition and action, and shows up as an ***affordance space*** (Brincker, 2014; Gallagher, 2015) a ***landscape of affordances*** (Rietveld and Kiverstein, 2014; Gallagher et al., 2017). Affordance space is determined by different elements that pertain to individuals such as their specific characteristics and experiences, where they are in their (physical and mental) development, and/or by the constraints determined by socio-cultural practices. The term “landscape of affordances” refers to the vast array of affordances that are available to the agent. According to Glăvenau (2012, p. 196), “creativity can be defined as a process of perceiving, exploiting, and ‘generating’ novel affordances during socially and materially situated activities.” The main point is how individuals perceive opportunities within the limitless number of affordances they encounter. This is particularly relevant to the study of any creative acts. Thus, Glăvenau (2012, p. 197) proposes a tripartite model to theorize creative acts that comprises the intentionality of the actor and the normativity of any cultural context (**Figure 1**).

Glăvenau (2012) underlines the importance of making visible these uninvented, unperceived, and unexploited affordances. The main question here is the relation to the norm; he associates creative expression with the exploration of a ‘terra incognita,’ i.e., of environmental affordances that were previously unavailable to the person. Using a case study based on traditional Easter eggs-decoration in an Orthodox community in Eastern Europe, he shows how transgressing the norm, without violating it, has a creative force as it makes more affordances visible, and therefore available, and eventually allows breaking some of these rules, even while still staying within the overall tradition.

This vision of affordances as enriched by ecological and socio-cultural psychologists fully aligns with the ecological view of language education (van Lier, 2004; Kramsch, 2008) and is relevant for conceptualizing the potential of plurilingualism. The role of perception (‘noticing’: Schmidt, 1990), key in language learning, implies active exploration of and ability to discriminate between environmental data and make sense of non-verbal cues. In a plurilingual approach, perception, exploration and action are crucial, as is the need to build upon different semiotic cues (i.e., co-textual and contextual linguistic, paralinguistic, and non-linguistic signs). The crucial question of the relation to the norm is reconsidered in a plurilingual approach, which encourages a flexible and creative attitude to norms as a way toward meaning-making, construction of knowledge and development of criticality.

## Linguistic Diversity and the Plurilingual Vision: Toward a Paradigm Shift

The term plurilingualism, introduced in the 1990s in a key document in language education, the *Common European Framework of Reference for Languages* (CEFR) (Council of Europe, 2001) and related studies (Coste et al., 1997/2009) newly conceptualized the implications of linguistic diversity. Plurilingualism has since become a pillar of the vision of language education in Europe, fostering rich debate and innovation (Piccardo, 2013, 2014; Grommes and Hu, 2014; Piccardo and Capron Puozzo, 2015). Plurilingualism is a notion broad and strategic enough to address the monolingual myth and a generalized monolingual disposition (Gogolin, 1994), with languages learned and taught in isolation, aiming toward the model of the ideal – and idealized – native speaker.

Alongside plurilingualism, that we defined at the beginning of the article, two other interesting notions stand out from a recent proliferation of related terms: *multi-competence* (Cook, 1991; Cook and Wei, 2016) which challenged the notions of ‘native speaker’ and of separation of languages in the brain, and *translanguaging*, which opened the door to the use of multiple languages in classrooms (Williams, 1996; García, 2009). Proliferation of notions concerning linguistic plurality (see Piccardo and North, in press) shows the need to reconceptualize language and language learning, challenging monolingual assumptions. The key distinction is between multilingualism and plurilingualism. In contrast to multilingualism, plurilingualism relies on complex cognitive processes, as does creativity. Complexity is the common denominator linking plurilingualism and creativity.

In the CEFR, multilingualism is defined as “the knowledge of a number of languages, or the co-existence of different languages in a given society” (Council of Europe, 2001, p. 4). In such a definition, no attention is paid to the relationship between languages either at the level of the individual or of the society. In plurilingualism, on the contrary, the principle of relationship is fundamental since an individual “does not keep … languages and cultures in strictly separated mental compartments, but rather builds up a communicative competence to which all knowledge and experience of language contributes and in which languages interrelate and interact” (Council of Europe, 2001, p. 4). To better clarify the nature of plurilingual competence, examples are provided which all stress the capacity for flexible and hybrid uses of languages. These include the use of more than one language or dialect in interacting with other people, through forms of code switching, code-mixing and translanguaging, i.e., ways of referring to alternating between and/or blending languages, language varieties and codes (for a fuller discussion see Piccardo and North, in press). But it also includes the use of paralinguistic features or a simplified code, leveraging international terms – or linguistic forms from other languages – in order to decode spoken or written texts.

Plurilingualism integrates the idea of imbalance, adopts a perspective of development and dynamism, and encourages risk-taking through a flexible and creative use of the language. “The plurilingual perspective centers on learners and the development of their individual plurilingual repertoire, and not each specific language to be learnt.” (Beacco et al., 2015, p. 23). Plurilingualism stresses the role of the user/learner as a holistic being acting socially, whose personality develops through complex interaction of his/her own entire set of resources: cognitive, emotional, linguistic and cultural. In fact:

“Language is not only a major aspect of culture, but also a means of access to cultural manifestations. […] in a person’s cultural competence, the various cultures […] to which that person has gained access do not simply co-exist side by side; they are compared, contrasted and actively interact to produce an enriched, integrated pluricultural competence, of which plurilingual competence is one component, again interacting with other components.” (Council of Europe, 2001, p. 6)

Thus, plurilingualism opens up a complex vision, where elements of a different nature inter-relate and influence each other. It also underlines interdependence between individuals and the social context. “As a social agent, each individual forms relationships with a widening cluster of overlapping social groups, which together define identity.” (Council of Europe, 2001, p. 1)

The new vision embedded in plurilingualism aligns with recent developments in the study of creativity, which is characterized by an increasing awareness of the complex nature of creative phenomena and the importance of the social and cultural dimensions, moving toward a paradigm of situated action and distributed cognition (Hutchins, 1995; Glăvenau, 2012).

## Toward a Complex Vision of Creativity

A full consensus on a definition of creativity has not yet been reached (Simonton, 2012) although, as mentioned above, a widely accepted definition of creativity comprises two-criteria: (a) novelty or originality and (b) utility, effectiveness, or appropriateness (Amabile, 1983; Simonton, 1999; Sternberg and Lubart, 1999; Lubart et al., 2003; Runco and Jaeger, 2012). More recently, two approaches have taken center stage: the cognitive approach, mainly focusing on individual mental representations and processes involved in the creative act (Ward et al., 1997), and a socio-psychological approach that considers both individual variables such as personality or motivation and the socio-cultural context (Sternberg and Lubart, 1995). The cognitive approach also started a discussion about the definitions of (Runco and Jaeger, 2012; Weisberg, 2015) and relationships between the constructs of creativity and intelligence (Jaarsveld et al., 2012, 2015; Plucker and Esping, 2015). Also, in the last few years, a broader socio-cultural perspective has appeared, which is characterized by a growing interest in the creativity of different communities and is nurtured through contributions from sociology and anthropology as well as cultural and socio-cultural psychology (Montuori and Purser, 1997; Tajfel and Turner, 2001; Jones, 2009; Glăvenau, 2010; Sawyer, 2012; Mouchiroud and Zenasni, 2013).

These developments result in a double definition: an individual one “creativity is a mental combination that is expressed in the world” (Sawyer, 2012, p. 7), and a socio-cultural one “creativity is the generation of a product that is judged to be novel and also to be appropriate, useful, or valuable by a suitably knowledgeable social group” (Sawyer, 2012, p. 8). The inextricability of the individual and social, personal and contextual dimensions was highlighted long ago (Runco and Jaeger, 2012, referring to Stein, 1953). The 1980s and 1990s have marked a move away from positivistic, person-centered, and univariate research toward socially oriented and dynamic conceptions of creative cognition, and systems-oriented research models (Glăvenau, 2013, referring to John-Steiner, 1992; Montuori and Purser, 1997; Friedman and Rogers, 1998; Jones, 2009; Sawyer, 2012).

**FIGURE 2:**
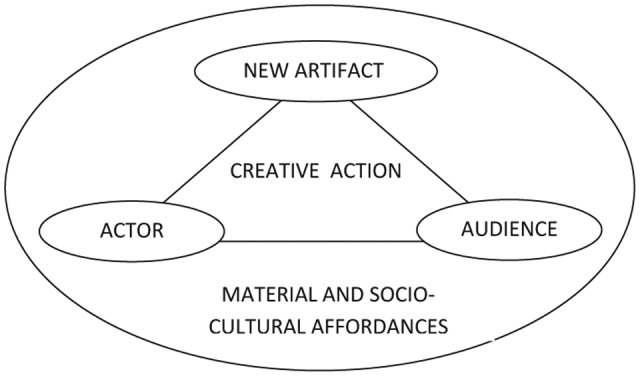
Five A’s framework of creativity (Glăvenau, 2013, p. 72).

New models have also been proposed to complement Rhodes’ (1961) four Ps of creativity: person, product, process and press. One tendency of these new models is to stress creativity’s multidimensionality as in the four-criterion construct: novelty, utility, aesthetics, and authenticity (Kharkhurin, 2014), or the need to radically change the lenses through which we theorize and study creative acts (Glăvenau, 2013). Glăvenau (2013, p. 70) stresses the growing importance of social, systemic, ecological, and cultural dimensions and proposes “a five A’s framework, that includes the following elements: actor, action, artifact, audience, and affordances,” whose relationship is shown in **Figure 2**.

This model discusses “creativity as a simultaneously psychological, social, and cultural process and [adds] to it a material dimension represented here by the creative use of affordances” (Glăvenau, 2013, p. 71). For Glăvenau (2013, pp. 71–72), “creative action emerges out of actor–audience relations that both produce and are mediated by the generation and use of new artifacts (objects, signs, symbols, etc.) within a physical, social, and cultural environment. In the end, this environment and its affordances are also gradually transformed by creative action.”

**FIGURE 3:**
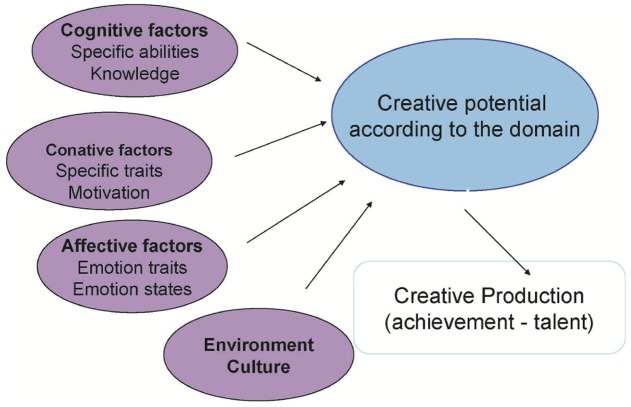
Multivariate model of creativity (Lubart et al., 2003, revised 2017 by the author, personal communication).

The multivariate approach (Sternberg and Lubart, 1995; Lubart et al., 2003; Lubart and Guignard, 2004), shown in **Figure 3**, is another useful model since it considers the dynamic interrelationship between a multiplicity of factors, thus aligning with DST.

According to Lubart, creativity depends on cognitive, conative, affective, and environmental factors:

• *Cognitive factors* include defining a problem, identifying relevant information, analogies, and comparisons, regrouping elements to create a new idea, generating alternatives (divergent thinking), self-evaluation, and flexibility.
• *Conative factors* include personality traits (e.g., perseverance, tolerance to ambiguity, risk-taking), cognitive styles, and motivation.
• *Affective factors* relate to the level of alert. Emotions, which come into play through past experiences, contribute to accessing concepts and to emotional resonance (creative associations, storing and retrieval of concepts).
• *Environmental factors* concern the contexts with which the individual interacts.

**FIGURE 4:**
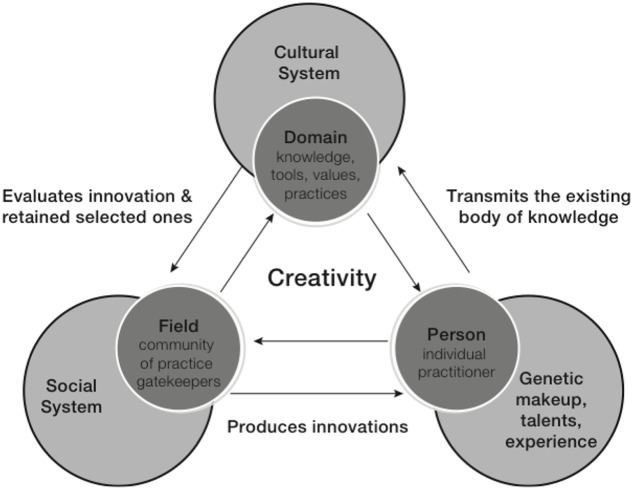
Systems model of creativity (Csikszentmihalyi, 1999, p. 315).

A third approach is the *systems model* of creativity (Feldman et al., 1994), shown in **Figure 4**, which represents creativity as resulting from the dynamic functioning of “a system composed of three elements: a culture that contains symbolic rules, a person who brings novelty into the domain, and a field of experts who recognize and validate the innovation” (Csikszentmihalyi, 1996, p. 6). Csikszentmihalyi (2015, p. 51) underlines that there is no starting point as “each of the three main systems – person, field and domain – affects the others and is affected by them in turn.” Each component is thus necessary for creativity but not sufficient *per se* to produce innovation. For Csikszentmihalyi (1996), creativity is the equivalent of the process that characterizes genetic modifications responsible for biologic evolution.

While the different models present several conceptual overlaps, for instance all models recognize the interplay of different factors in the creative act and the process involved in creativity, the various factors play different roles depending on the different angles taken. For example, the multivariate approach puts higher weight on the factors affecting the person while in the five A’s framework, actor (the person), artifacts, and audience (the environment) are equally weighted. Similarly, in the system model all elements seem to be equally important.

Now, as “all creativity is an emergent process that involves a social group of individuals engaged in complex, unpredictable interaction” (Sawyer, 2003, p. 19), these three models are key in conceptualizing creativity in a way that aligns with DST. According to the multivariate model, individuals are complex systems where several components interact (cognitive, conative, and emotional). In turn, the individual system is nested in a broader system, the environment, with which it interacts as described by the five A’s framework and the systems model. All models underline the interdependence of the different elements, actors and factors that come into play. We recognize here several characteristics highlighted earlier when introducing CAS. As we saw, one of the most interesting characteristics of CAS is emergence. The systems model aligns to a great extent with the emergentist vision which underlines how wholes have irreducible properties that cannot be fully understood or predicted by examining the parts alone. The five A’s framework stresses the interdependence of actor and audience, and how artifacts only exist within a social and material world and embody specific cultural traditions. Finally it makes us aware that what we perceive in our environment are affordances and not qualities as “we pay attention first to what can be done with an object rather than how the object is” (Glăvenau, 2013, p. 76).

We can consider creativity as an emergent property of the individual system who perceives affordances from the environment, which are opportunities for action on their part. Using these three models together, we can analyze each one of the three levels – individual, social and environment – and their interaction at the same time. This holistic perspective on creativity is essential if we want to capture the potential of plurilingualism and overcome the still very linear perspective of research on the impact of bi/multilingualism on cognition and creativity. Like in Second Language Acquisition (SLA) research, experiments which take isolated snapshots under laboratory conditions are at odds with the need to capture the situated complexity of the developmental process over time.

## A New Conceptualization of Languages and Language Learning Through Plurilingualism: Potential for Cognition

The rigid view of a machine-like brain with a fixed capacity where every component has a specific function is being replaced by a dynamic conceptualization where each function is not indissolubly linked to a cerebral area. The concept of neuroplasticity (Doidge, 2007) has brought about radical change (Li and Grant, 2016, p. 657). Furthermore, “the plasticity of the mind is embedded and inextricably enfolded with the plasticity of culture” (Malafouris, 2015, p. 351). In general, the brain is no longer seen as separated from the body or the environment, but rather as actively interacting with it in a dynamic process of reciprocal co-evolution over time. In particular, the brain of bilinguals and plurilinguals is no longer seen as the sum of monolingual brains, but rather as a unique, complex system (Bialystok, 2001; Perani et al., 2003).

Studies have increasingly shown that individuals with multiple languages have a cognitive advantage, precisely because they have constant practice at contrasting competing input and languages, and blocking irrelevant information (Abutalebi and Green, 2007; Thierry and Wu, 2007; Bialystok, 2009). A meta-analysis of 63 studies (involving a total of 6,022 participants) showed that “bilingualism is reliably associated with several cognitive outcomes, including increased attentional control, working memory, metalinguistic awareness, and abstract and symbolic representation skills” (Adesope et al., 2010, p. 207). Despite some critical voices (Paap and Greenberg, 2013; Paap, 2014; Paap et al., 2017), the body of research reporting a cognitive advantage related to bilingualism is impressive and growing (Bak et al., 2014). The topic remains controversial with inconsistent evidence concerning the difference between acquiring a second language in childhood or later in life, or with regard to the challenge of replicating existing studies. However, very few studies show any bilingual *disadvantage* (de Bruin et al., 2015).

We agree with Bak’s proposed ‘constructive skepticism’ approach to the issue: “It is constructive in assuming that both positive and negative results are genuine, but reflect possible differences in experimental designs, definitions of the phenomena in question, examined populations and the environment in which the research is being conducted” (Bak, 2016, p. 700). The context can have a significant effect depending on whether a monolingual mindset (strict separation of languages) or an openness to plurality dominates. For example, a context like the Indian subcontinent – where languages blend into one another and “overlap, interpenetrate, and mesh in fascinating ways” (Canagarajah, 2009, p. 9) even in the same speech situation – is conducive to a plurilingual mindset, which might help explain Alladi et al.’s (2013) claim regarding delays in the onset of dementia. By contrast, in a context like France – where such hybridity is frowned on – even language specialists with a high level of proficiency in two or more languages often retain a monolingual mindset and see themselves as non-plurilingual, because their proficiency in the different languages is not balanced (Carrasco Perea and Piccardo, 2009).

Furthermore, present studies do not generally align with a complex paradigm as they try to isolate discrete phenomena and investigate them in a linear manner. Learning a new language does not equate to mapping different words onto the same language-independent concepts, it also means creating new concepts and recalibrating existing ones (Athanasiopoulos, 2016). Individuals act within their social environments, which in turn are embedded in linguistically, socially and culturally defined broader configurations (Lantolf, 2011), two nested CAS, as envisaged by the DST. In addition, the vast majority of the studies are not concerned with the educational dimension and do not take account of the pedagogical environment.

As the CEFR explains, the acquisition of each language modifies the individual’s overall linguistic repertoire. While not each error is creative *per se*, some errors are proof of risk-taking and creativity in appropriating language, not just of interference. Language learning is seen as a non-linear process where linguistic competences and experiences come into play, alongside conditions and constraints, while accomplishing real-life tasks in a social context. Bilingualism as a springboard for learning additional languages is an example of such non-linearity, with synergies between languages. In this complex view, construction of language proficiency is improved by an autonomous reflexive attitude in which metacognitive and metalinguistic skills are enhanced through an active role. The CEFR definition of the learner as a ‘social agent’ emphasizes the interaction between individuals and society, and the agency exerted by each individual. Language learning is seen as an active, reflexive process in which new information is linked to existing knowledge rather than as habit formation, particularly in a plurilingual approach.

Plurilingualism thus challenges the dominant, monolingual vision that learning a language is putting a label on objects, events and ideas that exist independently from their linguistic denotation (Kramsch, 2009). The structure of a language affects the way speakers conceptualize the world (Whorf, 1956), which in turn has an impact on the cognitive process. The two basic cognitive mechanisms of creativity, juxtaposition of dissimilar and deconceptualization (Indurkhya, 2013), are perfect for plurilingualism: the former suggest juxtaposing linguistically and culturally dissimilar concepts or terms, thus potentially creating new meanings, insights and perspectives, the latter encourages moving away from existing conceptualization of objects and ideas through estrangement, associations and metaphors, thus helping us perceive differently and potentially gaining new insights. The plurilingual and pluricultural perspective paves the way to the development of ‘symbolic competence,’ encompassing a theoretical and practical ability and ‘spaces of possibilities’ “in the particularity of day-to-day language practices, in, through, and across various languages.” (Kramsch, 2009, p. 201). In this permeability between languages and cultures, aesthetic appreciation of the language, emotional involvement, exploration of both possible and invented meanings, play and creativity all co-exist. Thus, plurilingualism and creativity have a lot in common and the proposed theoretical framework has the potential to cast light on the links and synergies between them.

A compendium of research over the last 30 years published by the European Commission in 2009, the European Year of Creativity, to investigate possible links between multi/plurilingualism and creativity helps to frame our discussion. The researchers involved concluded that evidence clusters were ‘indicators’ that knowing multiple language results in people developing characteristics linked to ‘creativity.’ The clusters included: mental flexibility (the flexible mind); ability to cope with difficulties (the problem-solving mind); enhanced metalinguistic ability (the metalinguistic mind); better ability to learn (the learning mind); increased capacity for interpersonal communication (the interpersonal mind); reduction of age-related mental diminishment (the aging mind). These findings were complemented by the results of another study which proposed “that the cognitive connection between creativity and plurilingualism is the ability to perceive and produce new units of meaning … consequently, given high-level plurilinguals’ increased perceptual awareness, they are likely to gain new insights, create new analogies and experience creative moments in any domain where perception is at work” (Furlong, 2009, p. 365). The most recurrent transversal aspect of these characteristics is the capacity to cope with multiplicity and to adapt, building on existing internal and external resources. This relates again to the concept of affordances.

As Glăvenau (2012, p. 205) says, “to conceive of creativity in terms of affordances means to adopt a fundamentally **dynamic**, **relational**, and **action-oriented** approach to the phenomenon.” (emphasis added). These three characteristics are identical to those of plurilingualism as it has been theorized by the Council of Europe, whose plurilingual, real-world oriented pedagogy is entitled the ‘action-oriented approach.’ Embracing a plurilingual vision enables individuals to perceive and exploit affordances that they would not normally notice. In turn exploration would make those affordances increasingly visible thus expanding the ability to perceive and explore further – eventually more creatively – in a **dynamic circle**. As learners are social agents, this process is **relational** since it engages individuals within the material and social world in a reciprocal way. Finally, these social agents need **to act** within their environment, experiment with their linguistic and cultural resources and draw on these resources to engage with their physical and symbolic environment.

## Plurilingualism and Creativity: Enabling Possible Synergies

Plurilingualism differs from multilingualism in that it captures the dynamic, situated and complex relationship between languages within individuals’ linguistic and cultural trajectories and a proactive attitude toward linguistic and cultural plurality, with inventions of meaning (Zarate et al., 2008). In the discussion above I have highlighted several related elements: imbalance, risk-taking, out of the norm spaces, choice and agency among others. I will use now the theoretical models discussed earlier to connect these different elements and use the characteristics of *plurilanguaging*, “a dynamic, never-ending process to make meaning using different linguistic and semiotic resources” (Piccardo, 2017) to streamline discussion of the potential synergy between plurilingualism and creativity.

### Plurilanguaging Is a Cyclical Process of Exploring and Constructing

Learning a new language is characterized by phases of emergence. Individuals construct their learning in a recursive, complex way, adapting as a consequence of exposure to the language. However, learning a language is not just a one-way process since the user/learner acts on the environment while the environment is acting on him/her, and the two change over time as two nested CAS. Users can also influence language change on a larger scale, since languages are themselves CAS and continue to evolve without a fixed end-state (Larsen-Freeman, 2005). In a plurilingual view, every new language or dialect acquired builds on pre-existing knowledge and in turn shapes that knowledge as part of the individual’s plurilingual trajectory. So, within a DST perspective, this exploration and construction is a creative, emergent process enhanced by available linguistic and cultural resources. The models of creativity reviewed are relevant: factors presented by the multivariate model (cognitive, conative, emotional, environmental) are involved in the learning process; secondly, the individual agent sees his/her new learning filtered, accepted or modified by the relevant speech community in line with what the system model explains. This compares with cyclical processes of divergent and convergent thinking where the phase of hypothesis making, decoding and meaning-making is divergent and the phase of filtering and adapting to the speech community’s norms is convergent. This aligns with creativity, which is often seen as the result of multiple cycles of divergent and convergent thinking (Guilford, 1967; Kharkhurin, 2016). Talking about the cognitive mechanism underlying creativity among multilinguals, Kharkhurin points at evidence that multilingual practice facilitates both divergent and convergent thinking. In a plurilingual view, this alternation is purposefully sought, learners are encouraged and supported in a conscious process of divergent thinking and problem finding, and later are guided toward awareness of the norm in a convergent thinking process.

### Plurilanguaging Is an Agentic Process of Selecting and (Self)organizing

When an individual is engaged in a task, he/she only experiences affordances that he/she perceives as *relevant* to be invitations (Käufer and Chemero, 2015, p. 203). Thus, the individual, seen as a CAS, engaged in a process of language learning and use, responds to invitations by exerting his/her agency, focusing on the affordances (linguistic, cultural, cognitive, emotional, etc.) that are relevant to his/her learning process. Also, the individual acts within their material and symbolic world, drawing upon all their linguistic and cultural resources to mediate and (co)construct meaning. As a consequence, the entire nested system self-organizes and the individual reaches a new stage in the learning process, a new state of balance. However, in a plurilingual perspective and pedagogy, the relevant affordances multiply, consequently the need to make targeted decision increases. Furthermore, in a plurilingual approach uncertainty and elusiveness are not avoided, requiring both focused and lateral attention and thinking. All these aspects are conducive to creativity. Individuals with multiple languages are deemed potentially able to activate additional concepts due to the variation in the conceptual representation of translation equivalents, according to a language-mediated concept activation (LMCA) mechanism which stimulates divergent thinking (Kharkhurin, 2007, 2009, 2016). A plurilingualism paradigm involves making individuals aware of these aspects, thus further encouraging them toward divergent thinking and creativity.

### Plurilanguaging Is a Process of Dealing with Chaos

Systems are often in a state of unbalance as they undergo repeated cycles of order and disorder until they reach a new balance. However, as systems are dynamic and evolving, balance is only temporary. All learning, and particularly language learning, can be conceptualized following DST, but with plurilingualism, the notion of chaos is fundamental. For plurilingualism, imbalance is key (Puozzo Capron and Piccardo, 2014). It is only through positive acceptance of imbalance, awareness of the inevitably complex nature of language learning, that individuals are able to negotiate in-between spaces, construct and invent, and embrace change. The notion of ‘edge of chaos’ is a powerful metaphor: “a system at or near the edge of chaos changes adaptively to maintain stability, demonstrating high level of flexibility and responsiveness” (Larsen-Freeman and Cameron, 2008, p. 58). Plurilingualism stresses the fundamental role of dealing with chaos as a natural, positive state, where individuals feel free to make personal and creative use of all their linguistic and cultural resources. Research shows that individuals with multiple languages have a higher degree of tolerance of ambiguity (Dewaele and Wei, 2013), a fundamental trait of creativity (Vernon, 1970; Sternberg and Lubart, 1995; Zenasni et al., 2008). According to Lubart (1999) the routine ambiguity connected with using multiple languages, in which the same idea may take different nuances in different languages, can bring an advantage in divergent thinking.

### Plurilanguaging Enhances Perception in an Awareness-Raising Process

Perception awakens attention (Merleau-Ponty, 1962): we do not apprehend the world as something that exists separately, what we perceive *is* indeed the world. Furthermore, “perceiving, experiencing and the like are things that we *do*, not things that happen inside us” (Käufer and Chemero, 2015, p. 214). By embracing a plurilingual view, individuals experience a wealth of stimuli, as different languages encode concepts differently: “a plurilingual’s apprehension and perception of the world is likely to be expanded as a result of the multiple linguistic and pragmatic systems faced by him/her when engaging with interlocutors” (Furlong, 2009, p. 351). Embodied and situated cognition that align with DST and the theory of affordances help better conceptualize the functioning of the plurilingual brain. Rietveld and Kiverstein (2014, p. 326) “propose thinking of ‘higher’ cognitive capacities in terms of skillful activities in sociocultural practices and the material resources exploited in those practices. Skilled ‘higher’ cognition can be understood in terms of selective engagement—in concrete situations—with the rich landscape of affordances.”

The plurilingual view puts awareness at the core of the process. Selective engagement with a rich landscape of affordances requires awareness, which initiates change in the CAS and triggers further awareness of linguistic and cultural features, thus enhancing the entire learning process. Simple exposure to linguistic and cultural difference does not *per se* stimulate creativity (Maddux et al., 2010). To enhance cognitive flexibility and tolerance of ambiguity, personality traits relevant to creativity (Vernon, 1970; Lubart et al., 2003; Zenasni et al., 2008; Kharkhurin, 2016), metalinguistic/-cultural ability (i.e., a plurilingual/pluricultural approach) is necessary.

### Plurilanguaging Is an Empowering Process in Relation to Norms

Plurilinguals seem to have greater facility in perceiving unusual analogies and associations and an increased capacity for metaphorical thinking (Kharkhurin, 2012), a key to creative processes. “Where systems are stretched, where conventional rules are not upheld, where a point of criticality is reached, new forms emerge… New forms and patterns then become the resources of the community upon which members of the speech community can draw, exploit and reshape to populate with their own intentions and the affordances of the new context” (Larsen-Freeman and Cameron, 2008, p. 102). Embracing a plurilingual paradigm fosters a more flexible attitude toward norms, opening to hybrid spaces and the crossing of boundaries, both conducive to creativity (Maddux et al., 2010; Saad et al., 2013). As Furlong (2009, p. 356) reminds us: “Heightened perception of [*these*] boundaries and ‘in-between’ spaces … is crucial to enable the process of creativity.” As we saw when talking about affordances the ability to transgress norms is key to creativity too.

## Discussion and Conclusion: Toward a New Vision

Since Peal and Lambert’s (1962) seminal article claiming that bilingual children had better problem-solving and abstract thinking skills, higher concept-formation skills, and overall higher mental flexibility, the attitude toward speaking and learning multiple languages has changed. However, a change in mentality is slow: while we discuss whether or not bilingualism has positive effects, the monolingual vision continues to dominate education even in bi/multilingual contexts. Languages are generally taught separately following a rigid application of norms with no consideration for either the linguistic and cultural capital brought by learners or the potential of hybridity and cross-fertilization: a pedagogy of mono/multilingualism dominates.

The paradigm of simplicity and linearity is still pervasive both in education and research, with separate language curricula, parallel linguistic communities, and research regarding bilingualism as mere addition of two languages. The finding in some research on bilinguals that more than two languages shows no increase in cognitive advantage (Alladi et al., 2013) may be a consequence of the simple addition of languages in a linear paradigm, though certainly further studies would have to confirm this. Not only does the academic community have a tendency to disregard both the environmental impact in research on cognitive advantage (Bak, 2016) and the potential relationship between linguistic plurality and creativity (Kharkhurin, 2012, 2016), but also the way in which languages are used and taught and their position within individual linguistic and cultural trajectories and repertoires are neglected.

Beghetto and Kaufman (2014) propose some insights for a creativity-supportive learning environment: (1) incorporate creativity in your everyday teaching; (2) provide opportunities for choice, imagination, and exploration; (3) monitor the motivational messages being sent by one’s classroom practices; (4) approach creativity and academic learning as means to other ends, rather than as ends in themselves; (5) model and support creativity in the classroom. These insights fully overlap with prerequisites for a plurilingual pedagogy.

The change of paradigm from multilingualism to plurilingualism entails is a radical one that aligns with the characteristics of creativity. Creativity has a complex and dynamic nature just as plurilingualism has, as discussed above in the light of DST, the theory of affordances, and three complementary models of creativity. By clarifying what *plurilanguaging* implies, I have presented possible synergies between plurilingualism and creativity and discussed the potential of plurilingualism for enhancing creativity. Although no claim of a causal relationship can be made, the striking similarities between conditions conducive to plurilingualism and creativity deserve further consideration and investigation.

What is crucial is to provide favorable conditions for plurilanguaging to increase the chances that creativity is enacted. Embracing plurilingualism can initiate change from the tiniest to the broadest scale, from helping individuals see the interconnections between language systems and ‘discover’ their full repertoire, thus liberating their plurilingual self (Piccardo, 2013), to empowering them in perception, awareness and active exploration of linguistic and cultural diversity, hybridity and interconnections. And this change is incremental – another similarity with creativity as the mini-c type of creativity can be encouraged by teachers, parents, and mentors (Kaufman and Beghetto, 2009).

Second language learners can bring a number of advantages to classrooms and not just challenges. At the same time “it remains unclear how, in practice, second language learners and their instructors may capitalize on these advantages. Further work investigating the cognitive correlates of bilingualism within educational contexts is required to clarify this issue” (Adesope et al., 2010, pp. 230–231). One such advantage may be enhanced creative potential. However, this does not happen by itself when languages are juxtaposed. Plurilingualism may be a catalyst for creativity but, like creativity, requires nurturing in education. Emergence happens at various levels within the CAS, involves nested systems interacting, and requires time and a certain level of complexity (Corning, 2002). If the different characteristics of plurilanguaging I listed are not encouraged and nurtured, the different CAS involved (individuals, educational institutions, society) will not experience phenomena of emergence nor perceive affordances, thus missing opportunities for creativity.

## Author Contributions

The author confirms being the sole contributor of this work and approved it for publication.

## Conflict of Interest Statement

The author declares that the research was conducted in the absence of any commercial or financial relationships that could be construed as a potential conflict of interest.
